# Current Stem Cell Biomarkers and Their Functional Mechanisms in Prostate Cancer

**DOI:** 10.3390/ijms17071163

**Published:** 2016-07-19

**Authors:** Kaile Zhang, Shukui Zhou, Leilei Wang, Jianlong Wang, Qingsong Zou, Weixin Zhao, Qiang Fu, Xiaolan Fang

**Affiliations:** 1 The Department of Urology, Affiliated Sixth People’s Hospital, Shanghai JiaoTong University, Shanghai 200233, China; great_z0313@126.com (K.Z.); 2005507098@163.com (S.Z.); zou_qingsong@126.com (Q.Z.); 2 Wake Forest Institute for Regenerative Medicine, Winston-Salem, NC 27101, USA; wezhao@wakehealth.edu; 3 VIP Department of Beijing Hospital, Beijing 100730, China; luckyleileiaaa@sina.com; 4 Urology Department of Beijing Hospital, Beijing 100730, China; wjlspplaaa@sina.com; 5 Department of Cancer Biology, Wake Forest University School of Medicine, Wake Forest Institute for Regenerative Medicine, Winston-Salem, NC 27101, USA

**Keywords:** prostate cancer, cancer stem cell, stem cell biomarker

## Abstract

Currently there is little effective treatment available for castration resistant prostate cancer, which is responsible for the majority of prostate cancer related deaths. Emerging evidence suggested that cancer stem cells might play an important role in resistance to traditional cancer therapies, and the studies of cancer stem cells (including specific isolation and targeting on those cells) might benefit the discovery of novel treatment of prostate cancer, especially castration resistant disease. In this review, we summarized major biomarkers for prostate cancer stem cells, as well as their functional mechanisms and potential application in clinical diagnosis and treatment of patients.

## 1. Introduction

**Figure 1:**
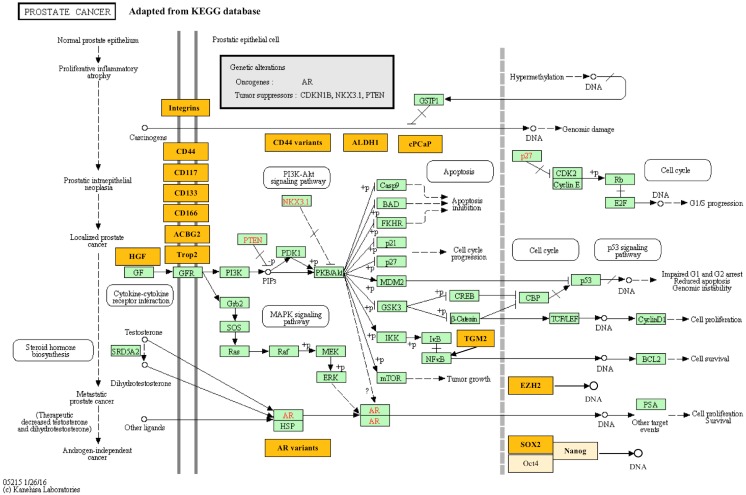
The mechanism and pathway map of the prostate cancer (modified based on KEGG database). Solid line between genes/molecules indicates direct regulation, while dashed lines indicates possible indirect regulation. Circle indicates a group of similar molecules (instead of a specific one). Biomarkers discussed in this review are highlighted in orange and in bold font, related molecules that are newly discovered are in yellow. Classic biomarkers included in KEGG prostate cancer pathway are highlighted in green. Key regulators in classical pathways involved in PCa are displayed in red (e.g., NKX3.1, PTEN, AR).

Prostate cancer (PCa) is the most common non-skin cancer in American men [1,2]. Standard PCa treatment includes radical prostatectomy, radiotherapy, chemotherapy and castration (either by drug or by surgery, mainly for androgen sensitive PCa), as well as immunotherapy and palliative therapy (mainly for castration resistant PCa (CRPC)). CRPC is responsible for majority of the PCa-related deaths [3], and currently there are two major hypotheses of CRPC carcinogenesis, the adaptive mechanism and the selective mechanism [4]. The adaptive mechanism suggests gene mutations in PCa cells (e.g., mutations of androgen receptor (AR)), dysregulated expression of genes, etc., contribute to CRPC development [5]. The selective mechanism, which is emerged in the last few decades, suggests that pre-existing castration-resistant subclones in primary PCa tissues and cancer stem cell selection dominates CRPC development (Figure 1) [6,7,8]. Recently, it has been suggested that stem-cell directed differentiation therapy could promote differentiation of cancer stem cells and sensitize them to anticancer drugs (such as synergistic androgen signaling blocking agents) [9].

Cancer stem cells (CSCs) were defined as cells with capacity of self-renewal and proliferation in cancer tissue [8]. Over years, scientists have been arguing about the origin of cancer stem cells. CSCs were suggested to originate from mutated normal stem cells, from mutated progenitor cells in the process of differentiation which re-gains the characteristics of stem cells, or from mature cells that re-acquired self-renewal ability [10]. Various cell surface markers were used to isolate CSCs, whose proliferative potential was verified by in vitro andin vivo assays (Table 1 and Table 2). This review summarizes recent research progress of current stem cell markers in PCa.

## 2. Integrins

Integrins are a family of transmembrane receptors known to participate in cell-cell adhesion and cell-surface mediated signaling, serving as bridges for cell-cell and cell-extracellular matrix (ECM) interactions [11]. Integrin could interact with specific ligands to transfer signals through cell-cell or cell-ECM interactions and stimulate expression of downstream target genes. Integrins were generally overexpressed in PCa [12,13]. In PCa, expression of α2-integrin and EZH2 is observed in a small fraction of cancer cells, which is supportive for their role as stem cell marker [14]. α2β1 integrin plays an important role in epithelia-stroma interaction, which is suggested to contribute to selective bone metastasis [12]. In the meantime, it could be a new marker to screen for prostate stem cells. Collins et al. [15] has discovered that the prostate stem cells expressing α2β1 integrin locate at basal epithelial layer. Approximately 1% of basal cells examined by confocal microscopy were integrin positive, and these cells could be isolated directly from the tissue on the basis of rapid adhesion to type I collagen. This isolated cell population displays basal cell phenotype, marked by expression of CK5 and CK14 and lack of expression of differentiation-specific markers (such as prostate specific antigen (PSA) and prostatic acid phosphatase (PAP)). These prostate stem cells could be cultivated in vitro and display much greater capability to form colonies in vitro (comparing to total basal cell population). When α2β1 overexpressing cells and stromal cells were transplanted subcutaneously into nude mouse, they could form structure of normal prostate gland prostate-specific differentiation [15].

Microarray experiments performed by several independent groups found that Integrin-α6 (also known as CD49f) is consistently overexpressed in hematopoietic, neural, and embryonic stem cells, and it is suggested as an effective cell stemness marker [16]. It has been used for characterization of prostatic progenitor cells [17,18], and was suggested as an emerging biomarker for PCa evaluation [14,19,20].

## 3. CD44

CD44 is a single-pass type I transmembrane protein and an important cellular adhesion molecule related to signaling to extracellular matrix. CD44 was considered as a marker of cancer stem cells from many organs including prostate [21,22,23]. It was located extensively on cell membrane and is important for cell adhesion and signal transduction. It was reported that CD44 positive cells from primary prostatic tumor tissues possess cell stemness [24]. Molecular studies demonstrated that CD44^+^ PCa cells retain certain intrinsic properties of progenitor cells [25]. CD44^+^ cells express high levels of stemness genes including *Oct-3/4*, *Bmi*, β-catenin and Smoothened (*SMO*) [2,26,27]. Kasper et al. discovered that in PCa cells (such as LNCaP, DU145 and PC3), CD44 positive cells had much greater proliferative capability than CD44 negative cells [28]. Van et al. isolated the DU145 cells from CD44^+^ and CD44^−^ Cells and tested the gene expression of stem cells by RT-PCR. Low expression of luminal cell markers (e.g., CK18) and AR were observed in CD44^+^ cells, whereas the genes highly related to stem cell proliferation and differentiation were overexpressed [29]. Recently, CD44 expression level was reported to be correlated with PCa grade in prostate biopsy samples [30], and proteomics analysis showed that CD44^+^ cells had positive correlation with genes related to cancer proliferation and metastasis [31]. However, Ugolkov discovered that expression of CD44 and Oct4 were observed in large populations of benign and malignant cells in the prostate, which is somewhat contradictory to the definition of stem cells as a small fraction of the total cell population [32]. Their results suggested that combined expression of embryonic stem cell markers EZH2 and SOX2 might be used to identify potential cancer stem cells as a minor (<10%) subgroup in CD44^+^ prostatic adenocarcinoma cells [32].

Recently, quite a few thorough analyses have been done on CD44 isoforms that are generated through alternative splicing of CD44 precursor mRNA. Those CD44 variants function distinctly in PCa and might serve as independent markers comparing to total CD44 expression level. For example, CD44v2 correlated with a better recurrence-free survival rate in PCa patients and is underexpressed in metastatic PCa cell lines [33]. Another well-studied isoform is CD44v6, which is associated with PCa proliferation, invasion, adhesion, metastasis, chemo-/radioresistance, and the induction of epithelial–mesenchymal transition (EMT) as well as the activation PI3K/Akt/mTOR and Wnt signaling pathways, and CD44v6 expression was closely associated with conventional prognostic factors and is identified as significant predictor for biochemical recurrence in PCa [34,35]. CD44v7–10 were overexpressed in PCa, and knock-down of CD44v7–10 by RNAi would significantly decrease invasion and migration in PCa cells [36].

Taken together, those results demonstrated CD44 RNA isoforms, but not total CD44 protein, might serve as specific marker for prostate cancer stem cells, though total CD44 protein level might still serve as a stem cell marker for other types of cancers [29].

## 4. CD133

CD133 is a glycoprotein with five transmembrane domains, generally expressed in various stem cells and endothelial progenitor cells but not in mature endothelial cells [37]. CD133 has been widely used, usually in combination with other stem cell markers such as CD44 and α2β1 integrin, to isolate cancer stem cells from prostate tumors with different Gleason grade, including cells from both primary and metastatic lesions [38,39,40,41]. Approximately 0.1% of cells in any prostate tumor displayed this phenotype, though there was no correlation between the number of CD44^+^/α2β1hi/CD133^+^ cells and tumor grade [23]. In normal prostate tissues, CD133 expression was observed in both basal and luminal cells [42]. Although its expression in normal prostate tissue is pretty low, CD133 is usually overexpressed in inflammation cell population [43].

Prostatic basal cells could be enriched based on α2β1 integrin (hi) expression and further enriched for stem cells using CD133 in non-tumorigenic BPH-1 cells [44]. It is demonstrated that the tumorigenic potential did not reside in the CD133^+^ stem cells but was consistently observed in the CD133^−^ population [45]. These data confirmed that benign basal cells include cells of origin of prostate cancer and suggested that proliferative CD133^−^ basal cells are more susceptible to tumorigenesis compared to the CD133^+^-enriched stem cells. These findings challenged the current dogma that normal stem cells and cells of origin of cancer are the same cell type(s) [45]. Intensive studies need to be done to learn more about the role of CD133 in PCa origination.

## 5. ALDH1

ALDH1 was suggested as a stem cell marker for both normal and tumor tissues [46]. As a cytoplasmic enzyme, ALDH1 has multiple intracellular aldehydes which can be converted into carboxylic acids, and could be involved in intracellular degradation of cell toxic substances [47]. ALDH1 expression was reported to be correlated with tumor grade and prognosis in PCa patients [48]. Burger et al. [49] found that cells with high ALDH enzymatic activity have greater in vitro proliferative potential than cells with low ALDH activity. Similar results were observed in an in vivo prostate reconstitution assay [49]. Thus, ALDH enzymatic activity might be used as a functional marker of prostate stem/progenitor cells and allow for simple, efficient isolation of cells with primitive features. ALDH α2+/α6+/αV + CD44^+^ cells also displayed high colonization in vitro and highly invasive tumorigenesis and aggressive metastasis characteristics in vivo [50]. p63 cytoplasmic aberrance is associated with high ALDH1A1 expression, and it was found that cytoplasmic p63 levels were significantly associated with the frequency of proliferating cells and cells undergoing apoptosis in prostate cancers [51]. These components are suggested to have an important role in prostate cancer progression and may be used as a panel of molecular markers [52].

The aldehyde dehydrogenase enzymes are likely to protect stem cells by detoxification of cell toxic compounds, which indicates that ALDH1 might prevent prostate cancer stem cells from conventional chemotherapy attack, while effective inhibition of ALDH1 could enhance the chemotherapy efficiency. Thus, ALDH1 could not only be used as a prostate cancer stem cell marker for prognosis, but also as a potential drug target in cancer treatment.

## 6. ATP Binding Membrane Transporters (ABCG2, Also Known as Breast Cancer Resistant Protein or BCRP)

Studies have shown that prostate cancer contains side population cells (SP cells), which could be isolated by flow cytometry techniques based on behavioral characteristics of stem cells. SP cells have stem cell properties that are exclusively mediated by ABCG2. As a result, ABCG2 is considered as a marker of SP cells, as well as a cancer stem cell marker. 

ABCG2 is ATP binding membrane transporters, and is related to prostate cancer multi-drug resistance [10]. After castration, ABCG2+/AR− prostate cancer stem cells could be isolated from prostate cancer tissues, and it is suggested that ABCG2 expression might protect prostate cancer stem cells from castration, chemotherapy and hypoxic environment. ABCG2 has been suggested as a biomarker for treatments targeting on prostate cancer stem cells [53]. Interestingly, Patrswala et al. [54] found that ABCG2(+) cells could produce ABCG2(−) cells, and both types of cells have similar tumorigenicity and colony formation ability. 30% of human cancer cell lines (and more in the bone marrow) and xenografts contain 0.04% to 0.20% of SP cells (low but detectable), yet most of the primary tumor cells have only a very small portion of the SP cells, almost impossible to detect [55,56]. Giving the evidence that non-recurrent PCa samples presented relatively lower level of ABCG2, compared to both normal tissue and recurrent samples, it might be associated with chemo-sensitivity [57]. Whether ABCG2 could be used as a specific biomarker in PCa diagnosis and prognosis is still unclear and requires further research.

## 7. SOX2 and EZH2

SOX2 and EZH2 are essential for the development of human embryonic stem cells. SOX2 is a transcription factor and plays a key role in maintaining undifferentiated status and keeping self-renewal ability of embryonic stem cells [58]. EZH2 is critical for embryonic stem cells rebuilding and embryonic development. Studies show that they play a key role in prostate cancer stem cells [32]. Recently, SOX2 and EZH2 are also suggested as markers in malignant glioma patients [59]. Ugolkov et al. [32] analyzed expression of CD44, CD133, Oct4, SOX2 and EZH2 in benign prostate tissues, high grade prostatic intraepithelial neoplasia (HGPIN) and PCa tissues, and found that EZH2 and SOX2 were expressed in <10% of benign prostate tissue, HGPINs and prostate cancer. In addition, 82% (27/33) of SOX2+ prostate cancer cases were EZH2+ type, and 100% (33/33) of cases were CD44^+^. On the other hand, CD44 was found in 97% of benign prostate and HGPIN cases, and in 72% of prostate cancer cases. CD133 was found in only a small portion of PCa tissues (6%, 4/67). Oct4 expression was found to be closely correlated with benign and HGPIN, but not with PCa. It is believed that CD44 and Oct4 were expressed in most of benign and malignant prostate cells, which is not likely to be representative for a very small proportion of cancer cells (such as cancer stem cells).

## 8. CD166

CD166 is a newly discovered molecular surface marker of prostate cancer stem cells [60]. CD166 belongs to the Ig family of type I transmembrane proteins, which mediate cell-cell interactions, and have been used as prognostic markers for a variety of cancers [1]. CD166 was reported to enrich sphere-forming activity of WT LSC (hi) and Pten null LSC (hi), and enhance the sphere-forming ability of benign primary human prostate cells in vitro and induce the formation of tubule-like structures in vivo [60]. CD166 could be used to identify and isolate human, murine prostate cancer stem cells and hormone refractory prostate cancer [61]. CD166 protein level is upregulated in human PCa, especially in CRPC patients. Although genetic deletion of murine CD166 in the Pten null PCa model does not interfere with sphere formation or block prostate cancer progression and CRPC development, the presence of CD166 on prostate stem/progenitors and castration resistant sub-population of cells suggest that it could be a surface marker of cell stemness. It could be a potential therapeutic target for prostate cancer therapies, as reduced expression of CD166 might be able to interfere or reverse prostate cancer metastasis.

## 9. cPAcP

cPAcP is a prostate specific differentiation antigen. In PCa cells, decreased cPAcP expression is associated with androgen-independent cell proliferation and tumorigenicity as seen in advanced hormone-refractory prostate carcinomas [62]. It was demonstrated that HDAC inhibitor treatment could result in increased cPAcP protein level in cPAcP positive cells, increase androgen responsiveness, and exhibit higher inhibitory activities on AR/cPAcP-positive PCa cells than on AR/cPAcP-negative PCa cells. These data indicate that cPAcP has potential clinical importance serving as a useful biomarker in the identification of PCa patient sub-population suitable for HDAC inhibitor treatment [63,64].

## 10. Hepatocyte Growth Factor

It was found that prostate cancer stem-like cells (CSCs)/cancer initiating cells (CICs) express hepatocyte growth factor (HGF) and that the HGF/c-MET proto-oncogene product (c-MET) signal has a role in the maintenance of prostate CSCs/CICs in an autocrine fashion. Immunohistochemical staining of HGF was compared to biochemical recurrence after radical prostatectomy, and patients with PCa tumors exhibiting HGF positivity of 5% or more had a significantly shorter biochemical recurrence-free period than that of patients whose tumor HGF positivity was less than 5% (*p* = 0.001). In multivariate Cox regression, preoperative PSA and HGF positivity had the potential to be independent predictors of biochemical recurrence following prostatectomy [65].

## 11. Tumor-Associated Calcium Signal Transducer 2

Tumor-associated calcium signal transducer 2 (also known as Trop2) is a type I membrane glycoprotein which transduces intracellular calcium signal and acts as a cell surface receptor [66,67]. Trop2 is highly expressed in epithelial related cancers, and its protein level often correlates with poor prognosis [68,69,70,71,72,73]. Trop2 positive cells could be identified as a subpopulation of prostate basal cells with stem cell characteristics, and it has been used as an effective marker for isolation of basal prostate progenitor cells [74,75,76]. In prostate cancer, scientists discovered that Trop2 regulate cancer cell proliferation, self-renewal, cell-cell adhesion and metastasis through β-catenin and β1-integrin signaling pathways [77,78,79]. Interestingly, Trop2 expression in prostate cancer cells was regulated by energy restriction, glucose deprivation and methylation [80,81,82], making it a potential drug target in cancer treatment. Moreover, anti-Trop2 bispecific antibody was approved to effectively lead pre-targeted immunoPET and radioimmunotherapy of PCa in preclinical models, which significantly increased PCa related survival [83,84].

## 12. CD117

CD117 (also known as c-Kit) is a receptor tyrosine kinase protein, and has been used as an important cell surface marker to identify hematopoietic progenitors in bone marrow [85,86,87]. CD117 overexpression was observed in several types of solid tumors including prostate [88,89], and is correlated with the capacity of cell self-renewal and cancer progression [90,91]. Circulating CD117 positive cell percentage is correlated with cancer progression and PSA values in advanced PCa [92]. CD117 could be activated by its ligand, Stem Cell Factor (SCF), to promote bone marrow cell migration, tumor dissemination and potential bone metastasis [91,92,93,94].

## 13. AR Splice Variants

AR splice variants were found to promote EMT as well as induce the expression of stem cell signature genes [95]. Over 10 different AR splice variants were discovered in PCa cell lines, PCa xenografts and human patient samples, and a few of them were dissected to understand their functions in cancer progression [96,97,98,99,100,101,102,103]. More importantly, AR splice variants, such as AR-V7, were suggested to contribute to the drug resistance after suppression of AR signaling, especially in CRPCs [104,105]. High level of AR-V7 was observed in CRPC specimen, but rarely in hormone-naïve specimen [102]. It was suggested that transition from negative to positive status of AR-V7 might reflect the selective pressures on tumor, which makes it a dynamic marker for PCa diagnosis based on liquid biopsy samples, such as circulating tumor cells (CTC) [106].

## 14. TGM2

Transglutaminases are enzymes that catalyze the crosslinking of proteins by epsilon-γ glutamyl lysine isopeptide bonds. While the primary structure of transglutaminases is not conserved, they all have the same amino acid sequence at their active sites and their activity is calcium-dependent. The protein encoded by this gene acts as a monomer, is induced by retinoic acid, and appears to be involved in apoptosis. TGM2 expression is shown to negatively regulate AR expression and to attenuate androgen sensitivity of prostate cancer cells [107]. TGM2 activation of NF-κB expression induces NF-κB binding to DNA elements in the AR gene to reduce AR gene expression, and triggers epithelial–mesenchymal transition [107]. This suggests that TGM2-regulated inflammatory signaling may contribute to the androgen dependence of prostate cancer cells [107]. Thus, TGM2 is concluded as a cancer stem cell survival factor in various types of cancers, including prostate cancer [108].

## 15. Conclusions

Studies of prostate cancer stem cells have gained much progress in the past few years and numerous potential approaches were discussed for novel PCa treatment [109,110]. This review summarizes the major intracellular PCa stem cell biomarkers, including a few novel markers discovered recently. The normal or pathological process and potential drug response reflected by those biomarkers were discussed, which might help with early diagnosis, prevention, drug target identification, drug response evaluation and so on. With the progress in study of circulating biomarkers, we expect that more candidates would be identified to facilitate PCa biopsies, especially those soluble markers (circulating tumor cells (CTCs), circulating tumor nucleic acid (ctNAs), miRNA, lncRNA, exosomes, etc.) for liquid biopsies.

## Figures and Tables

**Table 1:** Summary of prostate cancer stem cell biomarkers based on location and function.

Biomarker	Transmembrane Protein	Glycoprotein	Enzyme	Transcription Factor	Extracellular Protein	mRNA
Integrins	Yes	-	-	-	-	-
CD44	Yes	-	-	-	-	-
CD133	Yes	Yes	-	-	-	-
CD166	Yes	-	-	-	-	-
Trop2	Yes	Yes	-	-	-	-
CD117	Yes	-	Yes	-	-	-
ALDH1	-	-	-	Yes	-	-
ABCG2	Yes	-	-	-	-	-
SOX2	-	-	-	Yes	-	-
EZH2	-	-	Yes	-	-	-
cPAcP	-	-	Yes	-	-	-
AR splice variants	-	-	-	-	-	Yes
HGF	-	-	-	-	Yes	-
TGM2	-	-	Yes	-	-	-

Trop2, tumor-associated calcium signal transducer 2; ALDH1, aldehyde dehydrogenase 1; ABCG2, ATP binding membrane transporters; cPAcP, cellular prostatic acid phosphatase; HGF, hepatocyte growth factor; TGM2, transglutaminase II; SOX2, SRY-box 2; EZH2, enhancer of zeste 2 polycomb repressive complex 2 subunit.

**Table 2:** Summary of verifying studies and possible pathways of prostate cancer stem cell biomarkers.

Markers	PCa Cell Lines	Primary PCa Tissues	Mouse Models	Possible Involved Pathway in PCa
Integrins	Yes	Yes	-	-
CD44	Yes	Yes	-	-
CD133	Yes	Yes	-	-
CD166	-	-	Yes	-
Trop2	-	-	Yes	-
CD117	-	-	Yes	-
ALDH1	Yes	-	-	-
ABCG2	Yes	Yes	-	-
SOX2	-	Yes	-	-
EZH2	-	Yes	-	-
cPAcP	Yes	-	-	-
AR splice variants	Yes	-	-	AR
HGF	Yes	-	-	AR
TGM2	Yes	-	-	NF-κB

PCa, Prostate cancer.
